# DISENTANGLING KNOWLEDGE AND BIAS: COMBATING AGEISM WITH THE REVISED FACTS ON AGING QUIZ

**DOI:** 10.1093/geroni/igz038.1703

**Published:** 2019-11-08

**Authors:** Jensen Davis, Linda Breytspraak, Jacob Marszalek, Joan McDowd

**Affiliations:** University of Missouri-Kansas City, Kansas City, Missouri, United States

## Abstract

The Facts on Aging Quiz (FoAQ) was developed in 1977 as a 25-item True/False test of knowledge about older adults. Since that time, it has been utilized in hundreds of studies involving clarifying misconceptions, measuring factual knowledge across different groups, and assessing bias toward older adults. The current study examines the psychometric properties of a revision to the FoAQ created in 2015 that modified the original items and added 25 more to better reflect contemporary aging research. Participants were sampled using Qualtrics and MTurk platforms and targeted to equally represent the following four age groups: 18-34, 35-49, 50-64, and 65 and older. Exploratory factor analysis (n=956) did not support a multi-factor structure, contrary to previous theories of it having cognitive, physical, societal, and psychological health factors. A single factor model was forced which contained 28 items that only accounted for 26% of the variance in scores. The reliability reached satisfactory levels in the younger three age groups with the 28-item version but remained inadequate among those 65 and older. Small associations with the Expectations Regarding Aging-12 and Aging Semantic Differential scales were observed. In its present format, the FoAQ is not sufficient for research use but remains a useful tool in provoking discussion about age bias and areas in which people of all ages lack factual information. Researchers suggest an expansion in response options and further clarifying the use of this instrument as a measure of knowledge or bias.

